# Propagation of amorphous oxide nanowires *via* the VLS mechanism: growth kinetics†

**DOI:** 10.1039/c9na00134d

**Published:** 2019-07-17

**Authors:** D. Shakthivel, W. T. Navaraj, Simon Champet, Duncan H. Gregory, R. S. Dahiya

**Affiliations:** Bendable Electronics and Sensing Technologies (BEST) Group, School of Engineering, University of Glasgow G12 8QQ UK Ravinder.Dahiya@glasgow.ac.uk; WestCHEM, School of Chemistry, University of Glasgow G12 8QQ UK

## Abstract

This work reports the growth kinetics of amorphous nanowires (NWs) developed by the vapour–liquid–solid (VLS) mechanism. The model presented here incorporates all atomistic processes contributing to the growth of amorphous oxide NWs having diameters in the 5–100 nm range. The steady state growth condition has been described by balancing the key atomistic process steps. It is found that the 2D nano-catalyst liquid and NW solid (L–S) interface plays a central role in the kinetic analysis. The balance between the 2D Si layer crystallization and oxidation rate is quantitatively examined and compared with experimental values. The atomistic process dependencies of the NW growth rate, supersaturation (*C*/*C*^0^), desolvation energy (*Q*_D_) barrier and NW diameter have been analyzed in detail. The model successfully predicts the reported NW growth rate to be in the range of 1–10 μm s^−1^. A novel seed/catalyst metal-based synthesis strategy for the preparation of amorphous silica NWs is reported. A nickel thin film on Si is used as a seed metal for the Au assisted VLS growth of silica NWs. The experimental results provide evidence of the creation of SiO under the given conditions followed by Si injection in the Au–Si nano-catalyst solution. The usage of seed metal was observed to reduce the growth temperature compared to the methods reported in the literature and obtain similar growth rates. The technique presented here holds promise for the synthesis of sub-100 nm diameter NWs.

## Abstract 2

The work reports the growth kinetics of amorphous nanowires (NWs) developed by Vapour–Liquid–Solid (VLS) mechanism.

## Graphical abstract

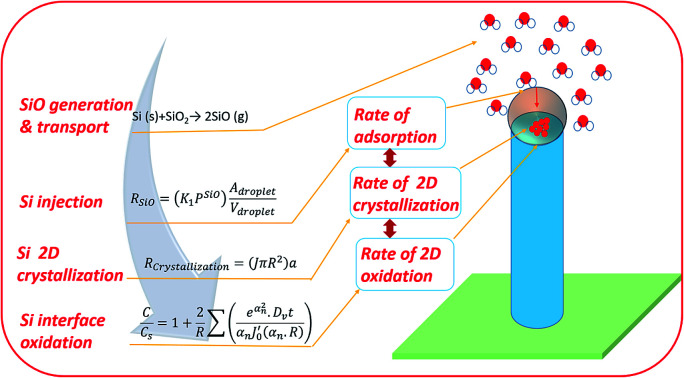

## 1. Introduction

Inorganic nanowires (NWs) with diameters < 100 nm are widely studied as they hold great potential for nanoelectronics,^1,2^ sensors,^3,4^ flexible electronics,^5–7^ energy generation,^8^ energy storage,^9,10^*etc.*, owing to their interesting physical and chemical properties.^11–16^ For example, the rapid change in the resistance of ZnO NWs with exposure to UV light makes them ideal for wearable dosimeters.^17^ Likewise, with photoluminescence (PL) bands in the range of 1.9–4.3 eV, the NWs of amorphous silica exhibit exciting optical properties, which could address the challenge related to blue light emission.^18,19^ Such properties are often exploited to develop novel sensors, IR detectors, efficient waveguides, *etc.* To this end, the NWs also require laborious tailoring of dimensions, growth processes, site-specific synthesis, *etc.* The task could be easier if there is a quantitative understanding of experimental conditions (*e.g.* temperature, pressure, *etc.*) during the NW growth process.

In this regard, the catalyst particle-assisted vapour–liquid–solid (VLS) growth mechanism has been extensively studied and there is a good understanding about the mechanism in the crystalline context.^6^ Surprisingly, the growth of amorphous NWs using the VLS mechanism has never been explained even if this method has been used to produce amorphous NWs such as silica (SiO_*x*_) and germanium oxide (GeO_*x*_).^20–22^ This is surprising because a large experimental dataset is available for VLS-synthesised silica NWs and, barring a small number of semi-quantitative models, the growth process for amorphous NWs remains relatively ambiguous.^23–25^ Herein we address this issue with studies focussing on the growth kinetics of amorphous NWs and an evaluation of the crucial parameters that influence the NW growth rates.

Silica NWs *via* the VLS method are typically grown at high temperature (>900 °C) by annealing Si sources (Si wafers, thin films and SiO powder) with nanoparticle catalysts (Au, Pt, Ni, *etc.*) under a flow of inert gas such as H_2_/N_2_ (forming gas), Ar or N_2_.^26,27^ The SiO vapours created *in situ* at these elevated temperatures act as the flux for NW growth.^28,29^ During the amorphous NW growth, these SiO vapours get adsorbed on Au catalyst particles and then Si species are injected into the bulk of the catalyst particles. Si injection leads to the formation of a nano Au–Si catalyst solution. When the Au–Si solution is supersaturated with Si, the growth of NWs is initiated at the catalyst–substrate 2D interface. This initiation of Si crystallization is attributed to the selective exclusion of oxygen molecules (or O^2−^ ions) from the Au–Si catalyst solution because of the poor solubility of oxygen at high NW growth temperatures.^30^ The growth of amorphous SiO_*x*_ (and GeO_*x*_) NWs starts when the oxygen atoms are directly incorporated at the catalyst-NW 2D interface and thereafter the coupled Si crystallization–oxidation process continues. Oxygen atoms get adsorbed at the vapour–liquid–solid triple phase boundary (TPB) and diffuse through the 2D interface for the oxidation process. The distinctive oxidation of Si crystals at this L–S interface is the key for amorphous NW growth. The interface-oxidation phenomenon is exclusive to NW growth, where oxidation occurs at the nano liquid–solid (L–S) interface,^31^ and is crucial in rationalising the VLS growth of amorphous NWs and their counterparts in crystalline metal-oxides (Al_2_O_3_, ZnO, *etc.*). The chemical composition of the catalyst solution (Au–Si) during this process also plays a crucial role in the synthesis of silica NWs.^22,24^

Taking the above established qualitative understanding as a basis, a kinetic model is presented here to explain the growth of amorphous NWs. The kinetic model is obtained from four atomistic steps:^32,33^ (1) Si species (*i.e.* SiO) generation and transportation, (2) Si incorporation into the catalyst particle, (3) Si crystallization at the interface, and (4) Si oxidation at the interface. Mathematical expressions have been developed for each of these steps to explain the steady state growth of NWs.^33,34^ The key feature of this work is the elucidation of the crystallization and oxidation processes occurring at the L–S interface. By using appropriate crystallization modes, the growth rate of NWs has been estimated with a range of kinetic parameters.^35,36^ The estimated growth rate has been found to be within the range of the experimentally measured values. The kinetic framework of the crystallization–oxidation phenomenon is verified by performing experiments under low oxygen partial pressure conditions. Si oxidation at the L–S interface is compared with the conventional vapour–solid oxidation of single crystal NWs.^37,38^ While the oxidation of crystalline Si NWs of 10–100 nm diameter takes from 30 min to 5 h, the direct growth of amorphous silica NWs of similar diameter can achieve lengths of several microns within 30 minutes. The kinetic model presented here shows a strong influence of the catalyst on the oxidation process at the L–S interface. The analogous case of amorphous GeO_*x*_ nanowire growth is also considered (Fig. S1†).

## 2. Experimental work

**Fig. 1:**
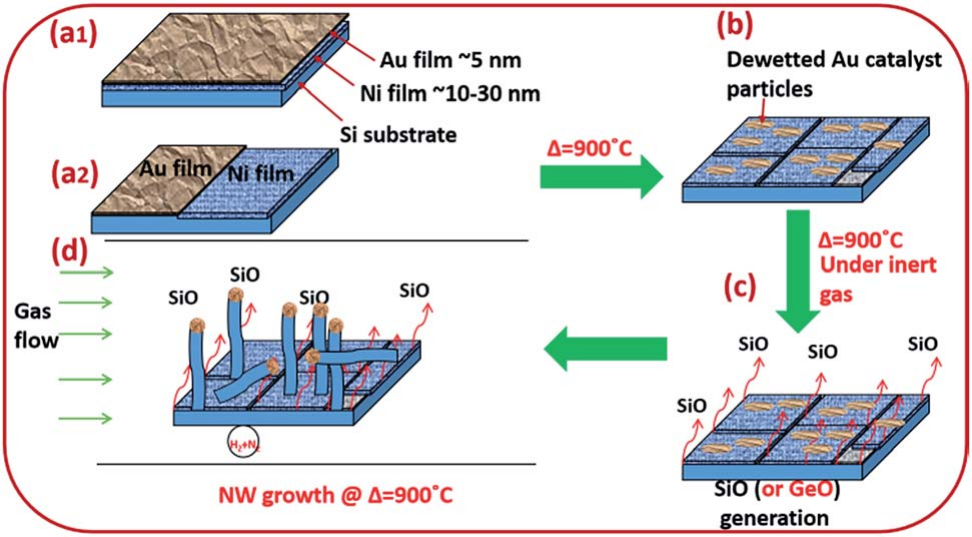
Schematic diagram of silica (or germania) NW synthesis. (a1) Si (or Ge)/Ni/Au stack configurations; (a2) Ni/Au bimetallic film deposited in parallel configuration; (b) de-wetted Au films, creating Au nanoparticles 5–100 nm in diameter; (c) generation of SiO gas; (d) site-specific growth of SiO_*x*_ or GeO_*x*_ NWs at Au nanoparticle sites.

Amorphous SiO_*x*_ and GeO_*x*_ NWs of diameter in the range of 5–100 nm were grown on silicon and stainless steel (SS) substrates using solid sources (Si wafers and Si and Ge thin films) to probe the atomistic steps of the VLS growth process. NW growth was performed in a resistance heating furnace under forming gas, 5% H_2_/N_2_ (99.99%, BOC), at 900 °C. Monocrystalline (100) p-type Si wafers were cleaned with acetone, isopropanol (IPA), and deionised (DI) water to remove surface contaminants. NWs were grown using bimetallic Ni/Au thin films to separate the monoxide vapours (SiO or GeO) and the catalyst particles from the solid source surface (Fig. 1). Two different Ni/Au bimetallic configurations have been prepared for NW synthesis (Fig. 1(a1 and a2)). Firstly, an Ni thin film typically of thickness 10–40 nm was deposited by e-beam evaporation. Subsequently, an Au thin film (3–5 nm in thickness) was deposited by sputtering onto the Ni surface (Fig. 1(a1)). Au films were annealed at 900 °C for 10 minutes under 5% H_2_/N_2_ to obtain statistically distributed catalyst nanoparticles of 5–100 nm in diameter (Fig. 1b). The Au film thickness plays a key role in determining the particle size distribution. Dewetted Au nano-particles (Fig. 1b) are produced by the film breaking at the grain boundaries where the grains are converted into particles.^39,40^ The average grain size in the Au thin films increases with thickness which has a direct impact on the resulting particle size distribution. The Ni thin film enhances SiO generation from the solid Si source (wafers and thin films) at 900 °C. The de-wetted Au nanoparticles act as a catalyst for NW synthesis by the VLS method. In the second Ni/Au configuration, the Ni and Au films were deposited in parallel on the Si surface to show that the SiO vapour is transported from the Ni/Si region to Au particles (Fig. 1(a2)). The flow reactor system used a quartz tube of 25 mm internal diameter. Si substrate samples of 1 cm^2^ area pre-deposited with Ni/Au films were placed in a quartz boat and loaded into the quartz tube reactor. In the case of the coplanar Ni/Au configuration, the substrate placement ensured that the Au layer was downstream of the Ni layer (*i.e.* that the gas flow would transport the anticipated SiO vapour towards the Au catalyst particles). The quartz reaction tube was sealed at both ends (gas inlet and outlet) with metal flanges equipped with O-rings and water cooling. The samples were heated to the synthesis temperature of 900 °C at a heating rate of 22 °C min^−1^ and were withdrawn after cooling to room temperature. SS deposited with Si films 1 μm in thickness were used as an alternative substrate and source for SiO vapour. The Si thin films were deposited on the SS substrate using PECVD (Oxford Instruments-Plasmalab 80+) followed by Ni and Au deposition as noted above for Si wafers.

Amorphous GeO_*x*_ NWs were synthesised under similar conditions to those described above except that Ge thin films (50 nm thick) were used by depositing Ge on an Si substrate by e-beam evaporation (ESI Section S1†). In additional experiments we explored the synthesis of silica NWs under low oxygen partial pressure conditions in a vacuum-sealed quartz tube. These experiments were performed to investigate the crystallization phenomenon at the catalyst/NW interface further. Low oxygen partial pressure was expected to prevent the oxidation of crystallized Si at the L–S interface. In a typical experiment an Si wafer coated with Ni/Au was sealed inside a quartz tube of 15 mm diameter under vacuum (10^−3^ Torr). The sealed tube was heated to 900 °C for 30 minutes to synthesize silica NWs.

SiO_2_ and GeO_2_ NWs synthesised *via* all the methods described above were characterised by Scanning Electron Microscopy (SEM-FEI Nova NanoSem 630) and Transmission Electron Microscopy (TEM-FEI Technai T20). NWs were mechanically removed from the substrate and dispersed in DI water for sonication. NWs were transferred to carbon-coated TEM grids for imaging. Bright field and HRTEM images were recorded under an operating potential of 200 keV.

## 3. Results and discussion

### 3.1. Silica NWs using Si wafers and thin Si films with Ni/Au stacks

**Fig. 2:**
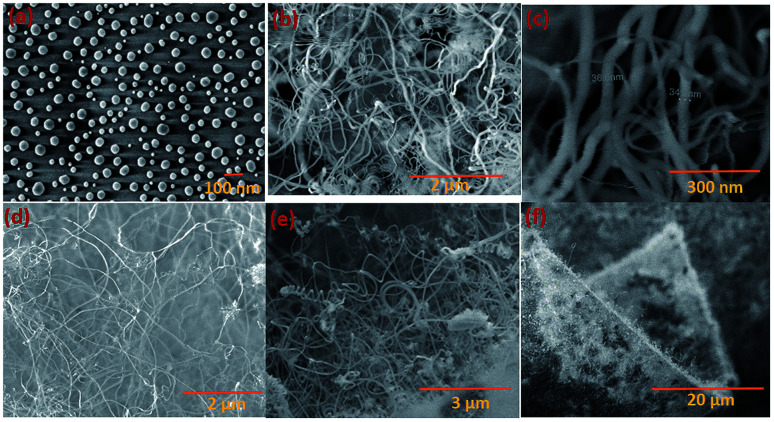
SEM images of silica NWs grown at 900 °C using Si wafers and Si thin films on an SS substrate. (a) Au catalyst nanoparticles prepared by de-wetting; (b and c) NWs grown using the Ni/Au catalyst and SiO source on an Si wafer; (d and f) silica NWs grown using PECVD of an Si film (1 μm thick) on the SS substrate. (f) SEM image showing an edge of the SS substrate covered with NWs.

**Fig. 3:**
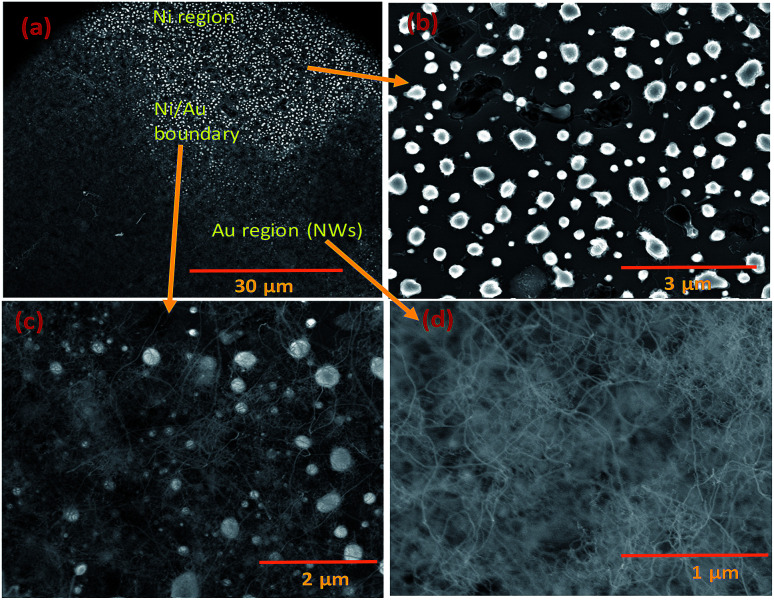
SEM images of Si NWs grown with Ni and Au in coplanar configuration. (a) Enlarged view shows both Ni and Au regions; (b) Ni particles without NWs; (c) region close to the interface between Au and Ni; and (d) silica NWs in the Au-deposited region.

Silica NWs were synthesised on Si wafers and SS substrates deposited with Ni/Au at 900 °C (Fig. 2 and 3). The first objective of these experiments was to verify that NW growth occurs only at the Au catalyst particle sites (from SiO vapours created *in situ*). De-wetting of the thin film was used to produce Au particles of 5–100 nm diameter at 900 °C (Fig. 2a). SEM images of the resulting silica NWs grown under these conditions are shown in Fig. 2b and c. The diameters of the NWs closely match with the dimensions of the Au nanoparticles, implying not only that growth occurs at the Au catalyst particle sites, but also that the diameter of the wires is expressly directed by the size of the Au particles (Fig. 2c). Based on Fig. 1 and 2, the SEM images indicate the two-fold utility of Ni films in the NW growth process: (1) promoting lower temperature VLS growth (at 900 °C and likely *via* SiO vapour generation *in situ*) and (2) providing physical separation between the NWs and the Si wafer to probe the growth kinetics. Previous studies have shown that SiO_*x*_ NWs are typically grown on Au-coated Si wafers at or above 1000 °C.^26,41^ Our experiments demonstrate, however, that growth of amorphous SiO_*x*_ and GeO_*x*_ NWs (ESI, S1†) is possible at lower temperature (<1000 °C). The use of Ni film has been extended to enable silica NWs to be synthesised on a stainless steel (SS) (3 mm thick, 304 L) substrate. A thin film of polycrystalline Si (thickness – 1 μm) was deposited using the PECVD technique on the SS substrate followed by Ni/Au stacking by evaporation. The polycrystalline Si film served as the Si source for SiO_*x*_ NWs. SEM images in Fig. 2d–f show SiO_*x*_ NWs grown on an SS substrate at 900 °C. The presented data suggest that NW growth using Si films on an SS substrate occurs just as it does on Si wafers *via* a VLS mechanism from SiO vapour. These results are especially promising in demonstrating the range of Si-coated substrates that could be available for silica NW growth. Clearly such substrate combinations could be selected depending on the foreseen applications. PECVD deposited Si films were observed to attach weakly to the SS substrate. A prolonged growth duration could result in a freestanding mesh of silica NWs.

The Ni and Au films deposited in a coplanar configuration on a 1 cm^2^ Si wafer by hard masking are shown as a schematic in Fig. 1a. The localization of an Ni layer (in its role as the initiator of SiO vapour formation) and Au layer as a catalytic surface for NW growth was tested with this arrangement and the results are shown in Fig. 3. The three unique surface regions so-created are shown in detail in Fig. 3b: Ni film fragments; Fig. 3c: the Ni–Au interface region and Fig. 3d: the Au particle region post-NW growth (which is restricted to the regions containing Au). The SEM images indicate that SiO vapour was likely predominantly created in the Ni region and was transported and adsorbed over Au catalyst particles. The Au catalyst particles were positioned upstream of the forming gas. The SiO vapours generated *in situ* would thus be transported from the Ni layer to the Au. As shown in Fig. 3b, the 10 nm Ni film fragments form discrete nanoparticles during the heating process. The thickness of the Ni films used was optimized to 10–40 nm. This range of thickness was necessary for NW growth (with additional observable variations in the Ni film morphology due to temperature effects). The 10 nm Ni film was broken into droplets (such as those shown in Fig. 3b) whereas 40 nm thick films stayed intact. Distinct regions over an area of the substrate could thus be discerned where the SiO flux had been transported from the Ni film to the regions containing the Au catalyst particles.

**Fig. 4:**
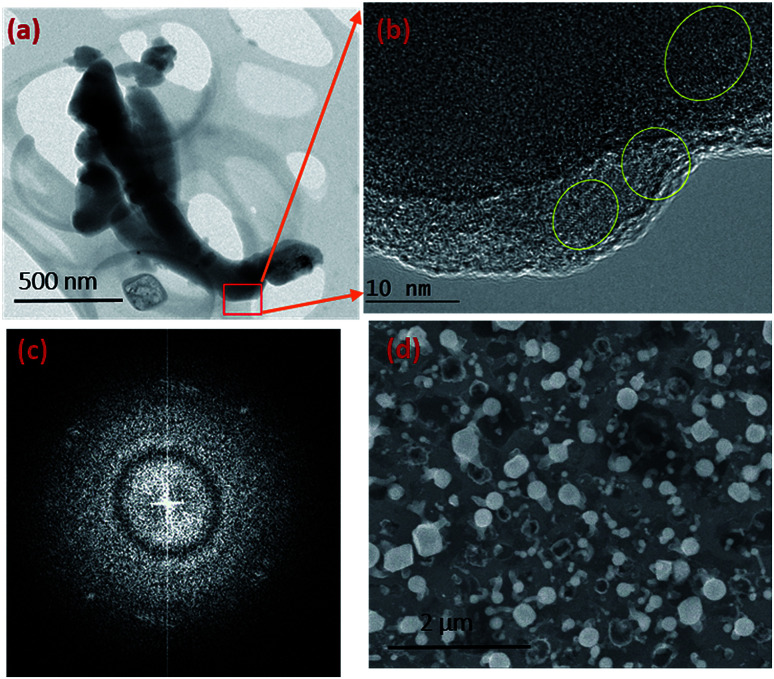
TEM and SEM images of silica NWs grown in a sealed quartz tube. (a) BF TEM image of NWs grown for 30 minutes. (b) Si crystallites encircled at the edges of NWs of image (a) where large parts are amorphous. (c) FFT pattern of the TEM image in (b) showing the diffraction spots arising from Si crystallites. The large diffuse rings prove that the NW is largely amorphous. (d) SEM image of the silica NWs over an area.

Given the experimental conditions used in this work, there are two reasons for the poor performance of Ni as a catalyst compared to the Au metal particles: (1) the Ni particles tend to form a thin oxide (NiO_*x*_) layer on the surface even under low oxygen and partial pressure conditions. This oxide layer prevents flux (SiO and GeO in this case) injection through the surface. The Ni metal is used for the growth of single crystal NWs (Si, Ge *etc.*) by the VLS mechanism under controlled ambient conditions, where pure H_2_ is used as the carrier gas. In this case, the formation of NiO is controlled by the lack of oxygen. (2) The Ni catalyst goes through many phase transformations compared to the Au, as shown in the Ni–Si^42^ and Au–Si^43^ binary phase diagrams below. The formation of an Ni–Si solution is feasible only above 1000 °C. However, as a noble metal, Au behaves as a catalyst above 363 °C without any problem of native oxide formation on the nanoparticle surface. Given the experimental conditions, SiO flux is preferentially absorbed by Au particles rather than by the Ni surface. The binary phase diagrams are presented in ESI Section S2.†Fig. 3b shows that a small number of NWs emerge from Ni particles. NW “bunches” from (larger) catalyst particles have been reported in the literature to grow from low melting metals such Ga and Sn.^22,27,44^ The existence of many of such NW “bunches” have been explained by invoking solid–liquid–solid (SLS) growth mechanisms, without considering the role of vapour generation. Depending on the experimental conditions, the silica NWs could be produced by any of the three mechanisms^45^ shown in Fig. S3-1.† The different mechanistic pathways described in the literature to explain these growth modes have been unified in this work in terms of a single kinetic framework. A common aspect of the growth modes shown in Fig. S3-1† is the existence of a L–S interface between the catalyst droplet and the emerging NWs. The creation mechanism of these various L–S interfaces may vary with experimental conditions. For example, in the case of bunched NWs, the relatively large catalyst particles precipitate NWs at various locations due the attainment of local supersaturation. The L–S interface acts as a sink for the supersaturated catalyst particles and the liquid catalyst solution continues to attract flux from the vapour for continuous NW growth. As shown in Fig. 3b, a relatively small number of NWs are located at the Ni particle sites. Given the relatively low temperature employed and the availability of Au particles in the close vicinity, the SiO flux is presumably preferentially ad/absorbed at the Au catalyst particle sites as compared to the larger sized Ni. The dissolved Si will tend to precipitate as crystallites at the L–S interface; we have studied this phenomenon in more detail by TEM as described more fully below (Section 3.1.1). It is proposed that elemental Si repeatedly crystallizes at the interface followed by rapid oxidation leading to prolonged NW growth. Analysis of the TEM micrographs of silica NWs grown under the described conditions did not reveal crystalline Si at the interface, however (Fig. 4b), this could be due to the relative timescales of Si crystallization and the subsequent oxidation and growth phenomena, which would be expected to perhaps range from milli- to micro-seconds. In view of the timescales of these processes, NW growth under low oxygen pressure conditions (Fig. 4) was attempted with the intent of separating and analysing the crystallization–oxidation processes.

#### 3.1.1. Silica NW growth under reduced oxygen partial pressure

The synthesis of silica NWs in a vacuum and under low oxygen partial pressure conditions has been reported previously.^46,47^ Under these conditions, the growth rate of silica NWs is up to five orders of magnitude lower than that in inert gas flow-based systems. Here, the Si substrates with Au/Ni (coplanar configuration) metal films were vacuum-sealed at a pressure of 10^−2^ mbar in an Ar atmosphere and heated at 900 °C for 30 minutes. SEM and TEM images of the resulting NWs are shown in Fig. 4a, c and d. Fig. 4d shows silica NWs less than 500 nm long formed after 30 minutes, which is roughly 50 times shorter than those grown under higher oxygen partial pressure conditions. The aim of the NW synthesis under low oxygen conditions is to probe the crystallization process. It will be shown in Section 3.3.2 that the crystallization and oxidation processes occur in the timescale of ms to μs. Hence, it is difficult to freeze the NW growth process to observe crystallization under atmospheric pressure conditions. Alternatively, silica NWs obtained from reactions in a vacuum-sealed quartz tube (Fig. 4a and b) indicate that crystallization of Si is a key step in the mechanistic pathway of silica NW growth. TEM samples were prepared by mechanically removing NWs from the substrate and dispersing the material in deionised (DI) water. The images (Fig. 4b) suggest that the NWs are largely amorphous but close examination of one of the NW edges shows regions containing crystalline lattice fringes (yellow circles in Fig. 4b). The measured values of the lattice spacings in these three regions are between 2.8 and 3.1 Å, which are close to the d-spacing for the Si (111) plane. Additionally, the FFT image (Fig. 4c) confirms the presence of crystallites through the diffraction spots. XRD analysis of the grown silica NWs was carried out to probe the crystallization process on a larger scale. The diffraction peaks predominantly show an amorphous structure with Au catalyst particles. The XRD pattern is presented in ESI S4.† The TEM analysis shows two important aspects related to amorphous NW growth: (1) oxygen partial pressure plays a crucial role in the creation of SiO species. SiO acts as a fuel for NW growth from vapour rather than from elemental Si; (2) growth occurs through crystallization–oxidation at the L–S interface. These processes will be evaluated as part of the kinetic model developed in the next section. The model explains all the atomistic processes involved in the amorphous NW growth process. The kinetic model will be used to estimate the growth rate of silica NWs with respect to growth temperature and catalyst particle size.

### 3.2. Development of the kinetic model

#### 3.2.1. SiO generation and transport

The gaseous SiO source molecules are generated *in situ*^28^ and absorbed by catalyst particles during synthesis of amorphous SiO_*x*_ NWs. The primary source of oxygen is inert gases, which typically contain 3–10 ppm of oxygen. These molecular species are usually produced under the flow of inert gases such as N_2_, Ar, forming gas (5% H_2_/N_2_) and pure H_2_. The pathways to produce SiO vapour are influenced by the thermal oxide thickness (*i.e.* the thickness of the oxidation layer – SiO_2_ – on the Si substrate), the partial pressure of residual oxygen in inert gases, the ambient pressure, temperature (>900 °C) and the presence of metal films (Ni, Au, Pt, *etc.*).^24,29^ SiO generation in the presence of pure H_2_ or forming gas can be expressed as:

1 H_2_ (g) + SiO_2_ (s) → 2SiO (g) + H_2_O (g)

This reaction is driven by H_2_ reduction of thermally grown SiO_2_ to create SiO vapours. Further, two additional pathways to produce SiO gaseous species are:

2 Si (s) + SiO_2_ (s) → 2SiO (g)

3
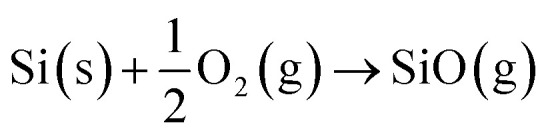


Reactions (2) and (3) describe the pathways of SiO creation that are possible under the flow of inert gases such as Ar, N_2_ and O_2_, where Si can react with amorphous oxide and O_2_ gas (at low partial pressure in the feed gas). However, metal films also influence the generation of SiO vapour. Results here and elsewhere show that deposited metal films of Au or Ni of thickness in the range of 1–20 nm break into droplets and can alloy with Si.^48^ The liquid droplets, Au–Si or Ni–Si could also contribute to the creation of SiO vapours, and Si atoms vaporizing from these liquid solutions would tend to produce SiO vapour. The contribution of SiO formed *via* this metal-assisted mode towards NW growth has not been reported before. This could be attributed to the low vapour pressure of Si on Au–Si under conventional experimental conditions. The evaporation of Si from metal melts (Au–Si and Ni–Si) contributes the lowest compared to steps (1)–(3). The possibility of SiO generation in a system with Ni and Au deposited in a coplanar configuration was studied in this work with the intention of identifying the contribution of each metal. Results suggest that Ni enhances SiO generation while Au catalyses NW growth. In summary, all of the three possible mechanisms defined by eqn (1)–(3) along with the alternative of Si vaporisation could contribute to creation of SiO vapour for NW growth.

#### 3.2.2. Rate of SiO incorporation (*R*_SiO_)

The injection of Si into the Au–Si catalyst particle occurs at the vapour–liquid (V–L) interface. The SiO species are adsorbed on the catalyst surface and chemically dissociated to allow for the transport of Si atoms into the droplet. The reaction equation for the chemical dissociation at the catalyst surface is given as:

4 2SiO (g) → 2Si (ads) + O_2_ (g)

Microscopically, the Au catalyst particles are converted into an Au–Si solution by dissolving Si from the substrate at and above a temperature of 363 °C, in accordance with the binary phase diagram. The rate of incorporation of Si into the droplet could be derived using Langmuir–Hinshelwood kinetics as:^34^5
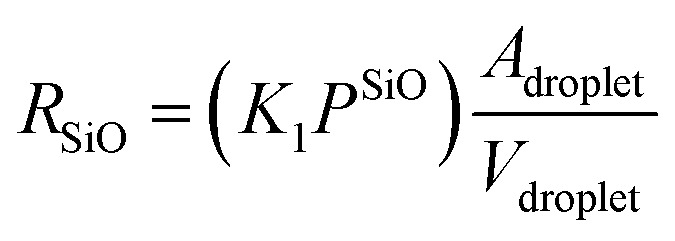
where *K*_1_ (m J^−1^ s^−1^) is the temperature-dependent rate constant associated with the cracking of SiO molecules on an Au–Si surface, *P*^SiO^ is the partial pressure of SiO under ambient conditions and *A*_droplet_/*V*_droplet_ is the dimensional constant of an Au–Si catalyst droplet (*A*_droplet_ and *V*_droplet_ are the surface area and volume of the droplet, respectively). The heterogeneous decomposition of SiO adsorbed over nanosized catalysts has not been studied previously. Moreover, no energy barrier values associated with SiO cracking on an Au–Si catalyst surface are available. The value of *K*_1_ could be crudely approximated by using the known expressions for the heterogeneous decomposition of Si-containing gaseous molecules such as silicon tetrachloride (SiCl_4_) and silane (SiH_4_) on a silicon substrate. In the case of SiCl_4_, *K*_1_ is given as:^49^6
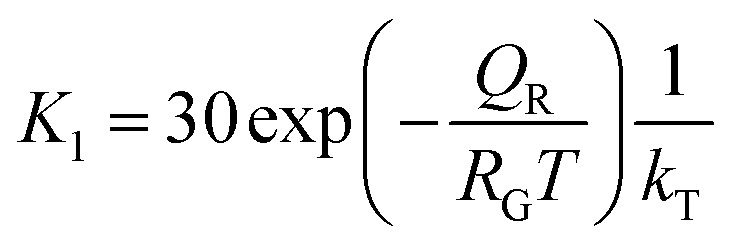
where *Q*_R_ (J mol^−1^) is the energy barrier associated with the dissociation of these molecules over Si or catalyst particles. The estimated value of *Q*_R_ for heterogeneous decomposition of SiCl_4_ was 100 kJ mol^−1^. Additionally, it is difficult to deduce partial pressure values of *in situ* generated SiO molecules.

#### 3.2.3. Rate of Si crystallization (*R*_crystallization_)

Si atoms are ejected out of the Au–Si liquid catalyst solution preferentially at the catalyst/solid interface upon attaining supersaturation. The saturation levels depend on the growth temperature and are quantified using conventional Au–Si binary phase diagrams.^35^ The layers of crystalline Si nucleate and spread at the L–S interface followed by dynamic oxidation to complete the amorphous NW growth. The oxidation process at the L–S interface results in amorphous silica similarly to thermal oxidation of single crystal wafers. Crystallization processes occur predominantly through two major modes, namely, layer by layer (LL) and multilayer (ML) modes.^50^ The LL mode proceeds with many Si nuclei forming at the interface and merging to form a complete crystalline layer before the next layer nucleates (Fig. S3-2†). Each layer may consist of single or multiple atomic layers of Si. The rate of nucleation and layer formation is highly dependent on the supersaturation of Si within the catalyst liquid. The rate of crystallization or wire growth is expressed as:

7 *R*_crystallization_ = (*J*π*r*^2^)*a*

where *r* is the radius of the interface, *a* is the height of the nucleated layer and *J* is the Si nucleation frequency per unit area. The nucleation frequency is expressed as:^34,50^8
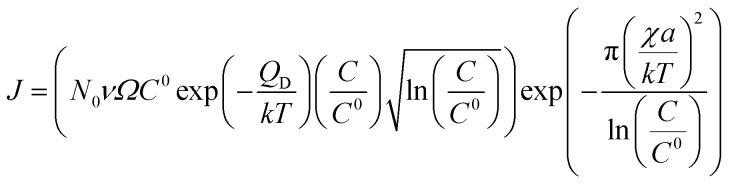
where *N*_0_ the number of atomic sites (10^19^ m^−2^) available for attachment, *ν* is the vibrational frequency (10^13^ s^−1^) of the atoms, and *χ* is the edge energy (10^−10^ J m^−1^) of the step. Here, step refers to the crystallizing layers of Si from the Au–Si catalyst melt. Depending on the supersaturation (*C*/*C*^0^) level, steps may consist of single to multiple layers of crystalline Si. These parameters are constant for Si nucleation from the Au–Si liquid solution. *Q*_D_ is called the desolvation energy and in this context is the barrier for Si atoms to detach from the Au–Si melt and to attach to the Si crystal. These data were deduced from incubation studies^35,36^ of NW nucleation from an Au–Si solution on a heterogeneous sapphire substrate. The value of *Q*_D_ can vary between 5 and 20 kT. Another influencing parameter of *J* is the supersaturation, *C*/*C*^0^, which could be estimated from binary Au–Si phase diagrams. Measurement of Si concentrations in nano Au–Si melt during growth is difficult and experimental data are unavailable. Post-growth analysis of catalyst particles would provide an estimate of the Si precipitated out of the droplets which is different from the supersaturated Si concentration during growth. Typical values of *C*/*C*^0^ have been identified in previous kinetic studies and verified by reported growth rates.^34^ The LL mode of nucleation and growth was experimentally verified with *in situ* TEM experiments for single crystal NWs of Si (and Al_2_O_3_) obtained using vapour–solid–solid (VSS) and VLS growth mechanisms.^31^ Additionally, it was proved that 2D crystal layers tend to nucleate along the edges of the L–S interface.

#### 3.2.4. Rate of oxidation (*R*_ox_)

The 2D interface oxidation process plays a central role in the conversion of crystalline Si 2D layers into amorphous silica NWs. It is clear that the process of oxidation at a liquid–solid interface is different when compared to the active oxidation of solid Si NWs.^38^ In general, depending on the temperature, a self-limiting oxidation occurs around the solid NW surface. For amorphous oxide NW growth to occur, oxygen diffusion at the interface must convert the nucleated crystalline Si layer into the amorphous oxide structure (Fig. S3-2†). Oxygen molecules cannot be transported through the catalyst particle due to poor solubility in the Au–Si solution.^30^ It has been verified experimentally that oxygen diffuses through the catalyst/substrate interface and reacts with the 2D crystalline layer.^31,51^ The transport of oxygen from the triple phase boundary (TPB) to form single crystal Al_2_O_3_ NWs was observed *in situ* in a TEM column by introducing oxygen gas.^31^ During silica NW growth, the presence of residual oxygen under ambient conditions ensures that the partial pressure (*P*_O_2__) is above the equilibrium value. This leads to the adsorption of a finite concentration of oxygen molecules along the TPB interface, as a first step towards the oxidation process, as depicted in Fig. 6. A saturated concentration of oxygen is required at the interface for Si oxidation to occur. Oxygen diffusion in the radial direction of a circular interface has been attempted in the past to obtain an estimate of the saturated oxygen concentration.^51^ Using this approach, the saturation fraction of the oxygen at the central point of the interface is expressed as:9
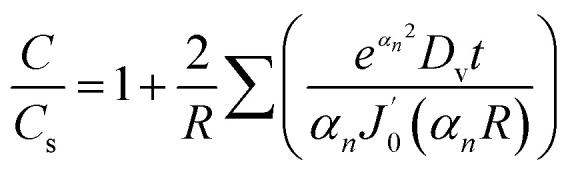
where *C* is the concentration of oxygen close to the TPB, *C*_s_ is the equilibrium oxygen concentration, *R* is the radius of the interface, *α*_*n*_ is the *n*th root of the equation, *D*_v_ is the diffusivity of the oxygen, and 
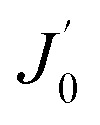
 is the derivative of a zero order Bessel function. The second term of this solution consists of a Bessel function. The time required to attain oxygen saturation could be obtained using eqn (9). The exact value of the diffusivity of oxygen at the Au–Si/silica interface is not available in the literature. The coefficient of interface diffusion is expressed as δ*D*_v_ and has value of 1.1 × 10^−19^ (m^3^ s^−1^) in the case of a liquid Al/Al_2_O_3_ interface.

#### 3.2.5. Steady state condition

The four atomistic processes which govern the kinetics of the amorphous NW growth occur sequentially in cycles during the steady state (Fig. S3-1†). The steady state condition is similar to a multistep chemical reaction where a balanced state is established to produce a final product.^52^ It is known that in a multistep reaction none of the intermediate steps individually control the whole process.^33^ Instead, the rates of intermediate reactions are equal and enable a steady yield of the final product. Similarly, the catalyst particles play an intermediate role in the NW growth process. There is continuous injection of atomic flux into the catalyst particle followed by ejection out of a droplet. So, at a steady state,

10 *R*_injection_ = *R*_ejection_

Generally, the ejection rate of an atomic flux is equal to the growth rate of the NWs. In silica (GeO_*x*_) NW growth, the ejection rate is equal to the rate of oxidation of silicon (germanium). The steady state condition for amorphous NW growth is expressed as:

11a *R*_injection(SiO)_ = *R*_ejection (crystallization)_ = *R*_oxidation_

The injection step, composed of vapour phase saturation from SiO sources, occurs *in situ* on the Si substrate. This gas phase concentration is highly dependent on the synthesis temperature which is varied between 900 and 1000 °C. The local partial pressure of SiO molecules on an Au–Si droplet dictates the rate of Si injection into the droplet. The kinetic parameters of the SiO adsorption on an Au–Si droplet are unavailable in the literature.

Hence, the current model will evaluate the steady state by considering the mechanistic processes that occur in the condensed phases. During the steady growth of NWs,

11b *R*_ejection (crystallization)_ = *R*_oxidation_

The assessment of this condition using the outlined equations provides insight into an amorphous NW growth process involving two elements (Si and O). The supersaturated concentration (*C*) of Si in the Au–Si catalyst particle plays a vital role in the whole growth process as the driving force for crystallization. Measurement of the Si concentration in a nano Au–Si catalyst is both challenging and tedious with existing experimental tools such as TEM-EELS. An estimation of the ratio (*C*/*C*^0^) between the actual concentration and the equilibrium concentration (*C*^0^) could be obtained from the binary Au–Si phase diagram. Estimates of the range of *C*/*C*^0^ are essential to evaluate the growth rate of NWs. The estimated growth rate of NWs will be correlated with the lateral oxidation rate. An understanding of the steady state condition will enable the relative significance of each of the atomistic kinetic parameters involved in the amorphous NW VLS growth process to be assessed.

### 3.3. Kinetic model: results and discussion

The growth model aims to elucidate how the growth rate of amorphous NWs grown using the VLS mechanism depends on each of the factors outlined above. Growth of silica NWs using an Au catalyst is considered in this model for a particle size/NW range of 10–100 nm diameter. The steady state growth rate of NWs was estimated for temperatures of 900 °C and 1000 °C and the values were compared with literature reports. This temperature regime was chosen given (1) that such moderate temperature ranges have produced silica NWs at reasonable growth rates experimentally and (2) the stability of Au–Si in the liquid phase over this range as reflected by the Au–Si binary phase diagram. The steady state condition (eqn (11a)) consists of two interrelated sequential mechanistic pathways leading to amorphous NWs. The Si layer LL mode crystallization rate (LHS of eqn (11a)) was calculated and equated with the oxidation rate. The effect of kinetic parameters associated with these atomistic processes has been evaluated.

#### 3.3.1. Rate of Si crystallization at the catalyst (Au–Si)/solid nano-interface

**Fig. 5:**
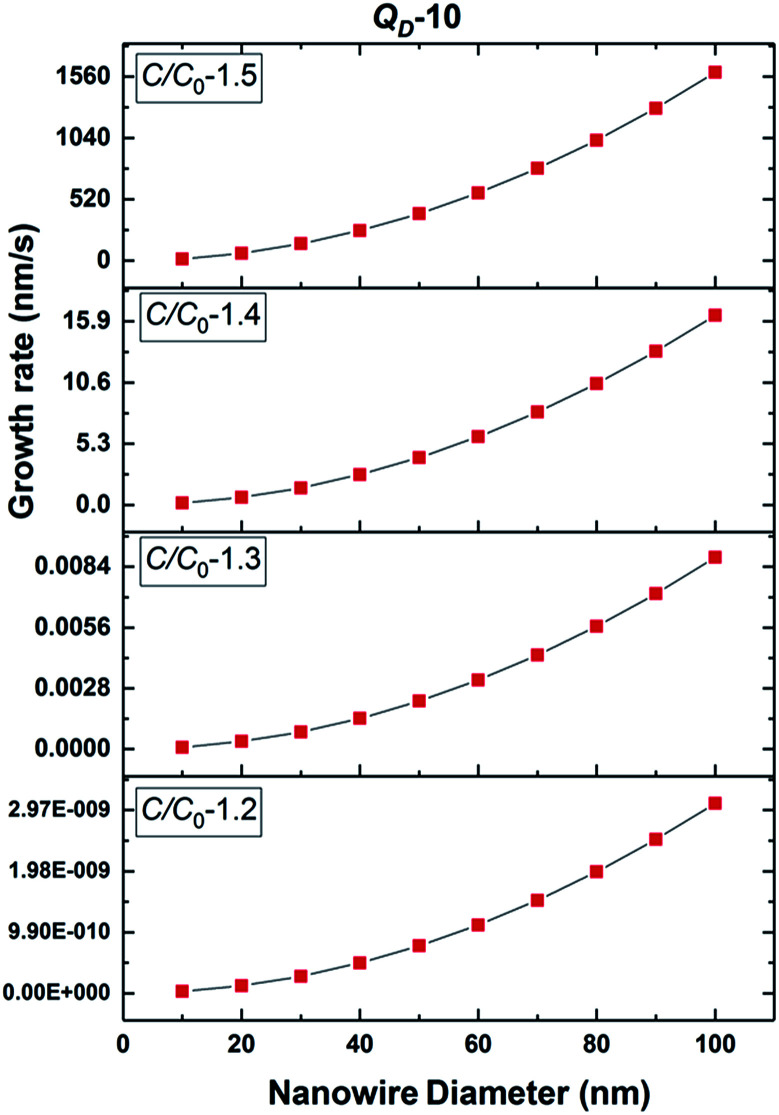
Estimated 2D Si crystallization rate with variation of supersaturation between 1.2 and 1.5 at 900 °C for *Q*_D_-10 kT.

The NW nucleation and growth process consists of an initial transient state followed by the steady state growth of the NW. The non-steady state regime begins with pure Au catalyst particles heated to growth temperatures (900 °C and 1000 °C). Initially, in addition to adsorbed Si atoms originating from the SiO vapour phase, Si atoms from the substrate are injected into the solid Au catalyst, which subsequently forms an Au–Si nano-catalyst solution. First nucleation of Si layers followed by oxidation occurs at the L–S interface which consists of supersaturated Au–Si (L) and the Si substrate (S). After the initial nucleation of the silica NW, growth proceeds at a steady state where the length of NWs is proportional to the growth duration.^32^ The equilibrium Si concentration (*C*^0^) in the nano-(Au–Si) catalyst solution depends on the temperature, and the Si crystallization occurs when the actual concentration (*C*) exceeds *C*^0^. The ratio, *C*/*C*^0^, is a sensitive parameter which plays a crucial role in determining the growth rate of SiO_*x*_ NWs. The theoretical variation of the crystallization rate at 900 °C with respect to the NW diameter can be calculated using eqn (7). Plots have been obtained for two different values of the desolvation energy, *Q*_D_, 10 kT (Fig. 5) and 5 kT. The complete set is presented in ESI Section S5 and S6.† These selected values are based on previous experimental work, which estimated *Q*_D_ to be in the range of 5–15 kT for the low temperature growth of Si NWs.^35,36^ Five-different *y*-axes show the variation of growth rate with *C*/*C*^0^ values ranging from 1.1–1.5 for each value of *Q*_D_.

**Fig. 6:**
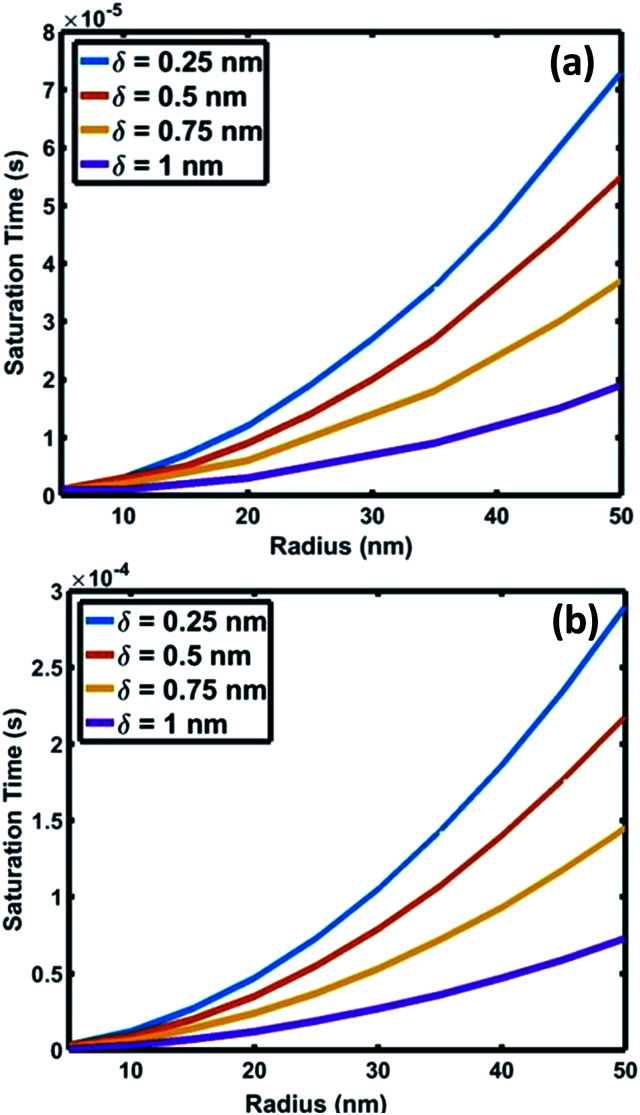
Variation of saturation time of oxygen as a function of interface radius for saturation levels (*C*/*C*_s_) of (a) 0.75 and (b) 0.999.

Based on these estimates, the Si crystallization rate (nm s^−1^) can vary by several orders of magnitude with an increase in supersaturation from 1.1 to 1.5. The experimental silica growth rates can be deduced to vary between 0.5 and 1 μm s^−1^ from an analysis of the literature.^26^ Given the curved and entangled nature of the amorphous NWs grown under the experimental conditions reported herein and the difficulty in obtaining precise lengths, these values are derived from approximate length estimates in the literature. Currently, an attempt has been made to deduce the experimental growth rate of silica NWs and compare the range with growth rates predicted by the kinetic model. NWs have been grown for different durations (few minutes to 60 minutes) and their cross-sectional length measurement (ImageJ^R^ software tool) has been used to obtain the NW growth rate. The measured average growth rate of NWs is found to be 650 nm s^−1^. Given the entanglement issue with amorphous NWs, this estimate could be the lower bound of the growth rate. However, this falls well within the range of the parameters, *C*/*C*^0^-1.5 and *Q*_D_-10, as shown in Fig. 5. This study helps to validate the developed kinetic model. Exact details of this characterization are presented in ESI Section 8 (S8†). As illustrated in Fig. 5, the crystallization rate falls in the range of 0.5–1 μm s^−1^ for *C*/*C*^0^ values of 1.4 and 1.5. The possible range of *C*/*C*^0^ values for the Au–Si binary system at 900 °C is between 1 and 2.3 with an equilibrium concentration of 42 at% of Si.^35,36^ In comparison, single crystal Si NWs that grow by the VLS mechanism using Si gaseous precursors (SiCl_4_ and SiH_4_) propagate at rates of 1–100 nm s^−1^,^32,43^ 5–10 times lower in magnitude than the growth rate of amorphous SiO_*x*_ NWs. The values of *C*/*C*^0^ have been estimated to vary between 1.1 and 1.3 maximum.^34^ The high growth rates could be attributed to localized processes that dominate the amorphous NW growth. The SiO molecular species created *in situ* during the growth process enhance the injection of Si into the catalyst particle as compared to the case of crystalline Si. In Si NW growth, Si vapour (from liquid SiCl_4_) and gas sources (silane) is diluted (1–10%) during supply. Under vapour phase transport conditions, more than 80% of these sources pass without reacting at the catalyst surface. As a result, the flux diluted 10–20% effectively contributes to growth. Compared to this scenario, silica growth under ambient conditions has a rich SiO source, which is probably one of the reasons for the higher order growth rate compared to that of Si NWs. This suggests that the steady flux injection–ejection is different for single crystal (Si) and amorphous (SiO_*x*_) NW growth processes. The estimates of the crystallization rate at 1000 °C are given in Section 3 of the ESI.†

**Fig. 7:**
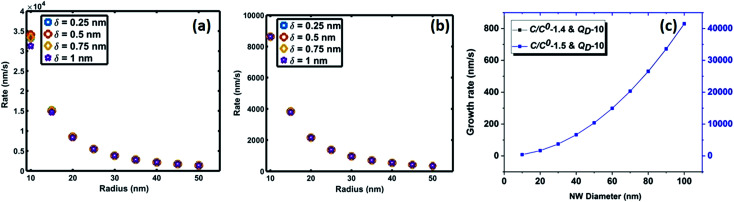
(a and b) Oxidation rate of crystallized 2D Si layers under two saturation conditions (0.75 and 0.99); (c) crystallization rate of Si 2D crystals at 900 °C.

#### 3.3.2. Oxidation rate of crystallized Si layers

The results of silica NW synthesis experiments suggest that the partial pressure of oxygen is high enough for O to be adsorbed and transported in the vicinity of the crystallized Si layers at the L–S interface. The expected limited solubility of oxygen in Au–Si droplets would restrict their adsorption to the TPB region. The adsorbed oxygen molecules would diffuse at the interface leading to the oxidation of the crystallized nano 2D Si layers (as depicted in Fig. S3-2†). With these assumptions, the interfacial diffusion of oxygen at the Au–Si/silica 2D nano-interface can be represented using eqn (9). Attainment of oxygen saturation at the interface and oxidation of Si are two sequential steps involved in the formation of amorphous SiO_*x*_ NWs. Here, one of the aims is to show that the process of progressive oxidation at the L–S interface is responsible for SiO_*x*_ NW formation and is entirely different from the active oxidation of a crystalline silicon NW that has already formed. In the proposed scenario, the crystalline Si layer is completely covered by the Au–Si liquid solution. Our analysis focused on finding (1) the time required for oxygen molecules to saturate at the nano-2D interface and (2) the rate of oxidation of the Si layers to form silica NWs. The dynamics of the oxidation process could be interpreted from calculations employing eqn (9) using a MATLAB code and subsequent plotting of the results.

The crucial parameter which determines the oxygen transport is the interfacial diffusivity (*D*_v_). Levi *et al.* defined an interface diffusion co-efficient, *δD*_v_ = 1.1 × 10^−19^ (m^3^ s^−1^), for oxygen diffusion at a liquid Al/Al_2_O_3_ interface at 1100 °C,^51^ where *δ* is the interfacial width. A range of *δD*_v_ values from 1 × 10^−19^ to 1 × 10^−21^ m^3^ s^−1^ is used in the current analysis of oxygen diffusion at the Au–Si/silica interface. Oxygen diffusion parameters at the Au–Si/silica 2D nano interface have not been studied through experiments or modelling. Fig. 6 shows the time required to attain oxygen saturation at the interface with a radius varying between 10 and 50 nm. The interfacial thickness of the Au–Si/silica interface, *δ*, is adjusted between 0.25 and 1 nm for oxygen saturation fractions of 0.75 (Fig. 6a) and 0.99 (Fig. 6b). The saturation time in the given diameter range varies from 0.01 ms to 0.3 ms for an oxygen saturation fraction of 0.75 and 0.999, respectively. Molecular O_2_ desorption should be minimal once oxygen steadily diffuses at the interface. The current scenario is different compared with the conventional Si wafer oxidation which occurs at the vapour–solid (V–S) interface. Oxygen desorption is common in V–S interface processes as the surface diffusion mechanism contributes significantly to the conversion of single crystal Si into amorphous silica. Under low oxygen partial pressures, this might lead to porous non-stoichiometric silica. The results of the silica NW characterization through EDX and EELS techniques showed that the Si : O ratio is 1 : 1.6.^23,25^ The oxygen saturation approximation (>0.75) considered in this model ensures that the silica NWs with Si : O stoichiometry falls in the measured range.

Fig. 7 compares the oxidation (a and b) and crystallization (c) rates under different growth parameters at 900 °C. These estimated crystallization and oxidation rates cover a wide range from 0.01 to >10 μm s^−1^ for the used thermodynamic and kinetic parameters. The oxidation and crystallization rates encompass the experimentally deduced silica growth rates in the range of 0.5–1 μm s^−1^. This shows that the oxidation rate of a crystalline Si layer is approximately the same order of magnitude as that of the NW crystallization process (Fig. 7c). This suggests that coupled dynamics of the processes at the interface decide the growth rate of amorphous NWs. It is evident that the saturation time is proportional to the interfacial area. The saturation fraction could influence the stoichiometry of the NWs. The overlap of the rate *vs.* radius plots in Fig. 7a and b shows that the interface layer width, *δ*, does not significantly influence the oxidation rate. The trends in the crystallization and oxidation rates are inversely related due to the different origin of these processes. The crystal growth of the Si layers is driven by existing supersaturation in the Au–Si droplet, whereas the oxidation occurs with a constant supply of oxygen molecules across the interface. The thickness of the growing crystalline 2D Si layer could vary from one monolayer to multiple atomic layers and this will depend entirely on the level of supersaturation. From the estimated growth rates (1–10 μm s^−1^), the time scale to grow a monolayer of Si (thickness 2.7 × 10^−10^ m) at the interface varies from 0.27 ms to 20 μs. The timescale, dimensions and operating temperature make it difficult to study such events using experimental tools. The oxidation process, which is modelled here as the concentration gradient-driven oxygen diffusion from the TPB to the central point of the interface, tends to create layers of oxygen molecules at the interface. The 2D Si crystallization occurs through mono- and poly-centre nucleation at the interface.^53^ Previous analysis considered that polycentre nucleation increases with the diameter of NWs and is attributed to the increased interfacial area.^51^ It is expected that the oxidation process could occur simultaneously during multiple nucleation events. The L–S interfacial oxidation process is very different compared to the V–S “active” oxidation of a pre-formed crystalline NW. Single crystal Si NWs have been oxidized by O_2_ gas at high temperature and modelled using conventional tools.^38^ The L–S interfacial oxidation timescale calculated here is 4–5 orders of magnitude faster than the V–S oxidation of single crystal NWs described previously.^37^ V–S NW oxidation occurs over a similar duration to that seen for bulk Si wet/dry oxidation (typically 30–120 minutes). The oxide thickness is limited by temperature. Similarly, when single crystal Ge NWs grown using an Au catalyst are heated between 325 and 450 °C under oxygen the product is amorphous GeO_*x*_ NWs.^54^ In this case, the Au catalyst promoted the formation of a Au–Ge eutectic solution, followed by the diffusion of Ge atoms across the catalyst particle. This would also suggest that 2D crystallization followed by oxidation is required for the formation of amorphous NWs.

## 4. Conclusions

Synthesis of amorphous silica and germanium oxide NWs in the diameter range of 10–100 nm has been studied experimentally, and a phenomenological kinetic model has been developed for their synthesis *via* the VLS mechanism. A steady state model was adopted to incorporate all mechanistic pathways contributing to the NW growth. Importantly, the analysis shows that elemental Si is injected through a nano Au–Si catalyst solution and that oxygen diffuses through the TPB interface for amorphous SiO_*x*_ NW propagation. The dynamic equilibrium between Si crystallization and oxidation is analysed with a range of thermodynamic (supersaturation) and kinetic parameters. The ongoing nature of Si injection, supersaturation, 2D crystallization and oxidation establishes a steady growth of NWs. The growth rate (0.5–1 μm s^−1^) of silica NWs was estimated for a diameter range of 10–100 nm and temperatures of 900 °C and 1000 °C. The experimentally measured growth rate of silica NWs is found to be 650 nm s^−1^, which closely matches with the growth rate predicted by the kinetic model. The growth rate is shown to be sensitive to the Si concentration in the Au–Si droplet. This model also shows that the oxidation occurring at the nano L–S interface is a different process from the V–S oxidation of crystalline NWs at high temperature. Oxidation of 2D Si layers is observed to occur in microseconds as compared to the V–S oxidation which requires hour timescales to complete. Synthesis of silica and GeO_*x*_ NWs was also performed to test the kinetic model. The role of SiO vapour in the NW synthesis and growth process was confirmed by physically separating Si/Ge sources from catalyst particles, and the growth of silica NWs under reduced oxygen partial pressures demonstrated that the crystallization process occurs at the L–S interface rather than *via* a post-growth V–S oxidation of crystalline elemental NWs.

## Conflicts of interest

There are no conflicts to declare.

## Supplementary Material

NA-001-C9NA00134D-s001
