# Supplementary material for: Propagation of amorphous oxide nanowires via the VLS mechanism: growth kinetics
Source: Nanoscale Adv. 2019 Jul 17;1(9):3568–78. doi: 10.1039/c9na00134d (PMC9418775; doi:10.1039/c9na00134d)
Supplement: NA-001-C9NA00134D-s001 [file NA-001-C9NA00134D-s001.pdf]

Electronic Supplementary Material (ESI) for Nanoscale Advances.
This journal is © The Royal Society of Chemistry 2019

Propagation of Amorphous Oxide Nanowires via the VLS Mechanism: Growth Kinetics

D.Shakthivel[1], W.T. Navaraj[1], S. Champet[2], D. H. Gregory[2], and R.S. Dahiya[1]

[1]Bendable Electronics and Sensing Technologies Group, School of Engineering, University of Glasgow, Glasgow, United Kingdom. [2]School of Chemistry, University of Glasgow, Glasgow, United Kingdom

S1: Amorphous germanium oxide NWs synthesis:

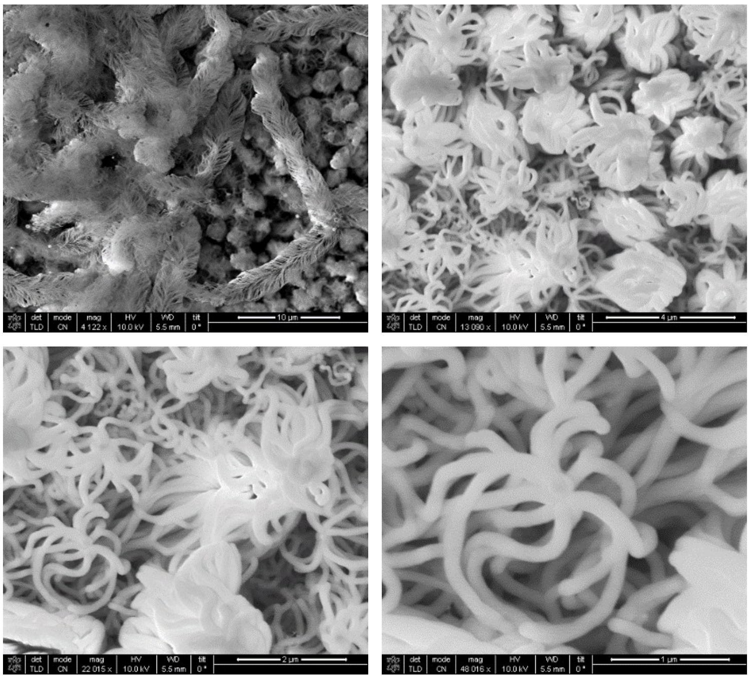

Fig.S1. Synthesis of amorphous GeOx NWs using Au assisted VLS growth mechanism at 900˚C. Crystalline Ge film (deposited by e-beam evaporation technique) was used as *in situ* Ge source.

Germanium thin films deposited using e-beam evaporation technique. Annealing has been carried out under the Ar gas at ambient conditions. Au nanoparticles are prepared using dewetting thin films. Nanoparticles and Ge thin films are separated which helps the Ge vapour to transport from Ge film and growth occurs at Au particles.

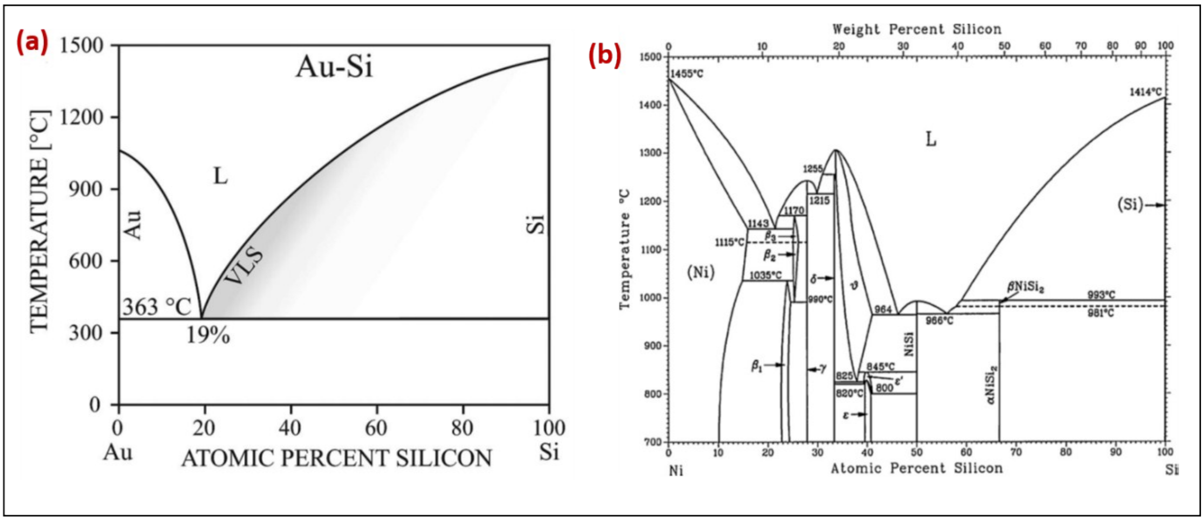

S2: Binary Au-Si [42] and Ni-Si [43] phase diagrams:

S3-1: Schematics of the VLS growth process of amorphous NWs growth:

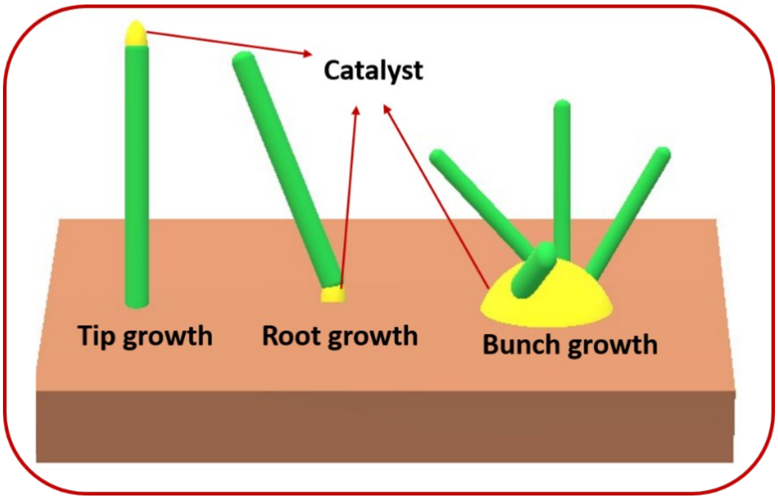

Fig.S3-1: Common VLS growth modes (Tip, root and bunch) of amorphous silica NWs.

S3-2: The atomistic processes outlined in sec. 3.2:

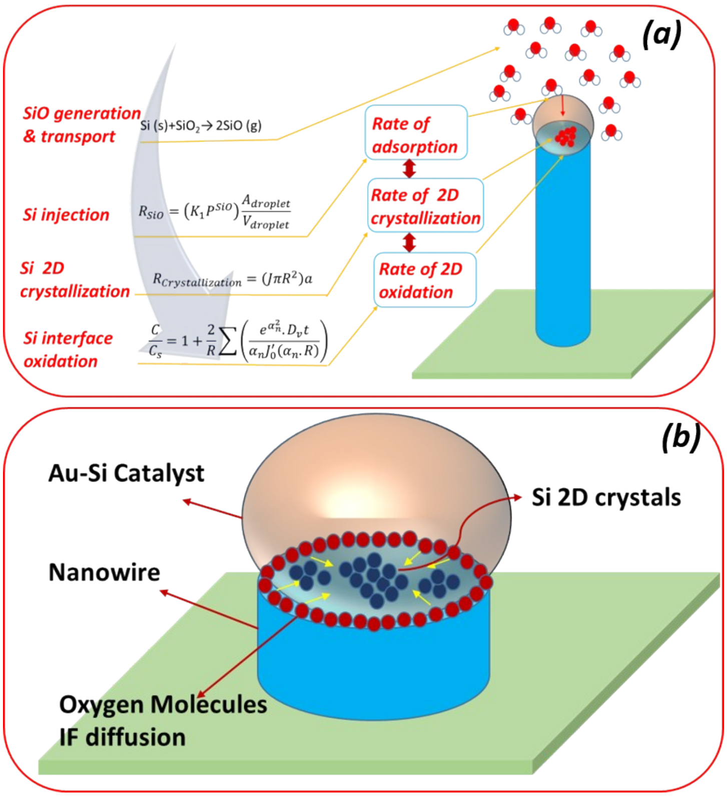

Fig. S3.2(a) Schematic illustration of the steady state growth of an amorphous SiO_x NW, indicating each of the contributing the atomistic process. (b) Schematic illustration depicting the 2D crystallization-oxidation phenomenon occurs at the L-S

S4: XRD analysis of the silica NWs grown using VLS mechanism

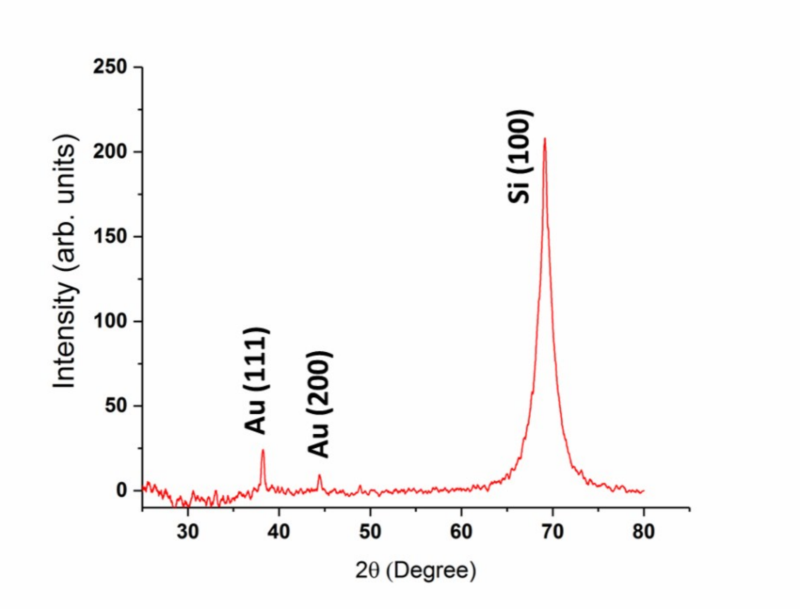

:

Fig. S4. XRD pattern obtained from silica NWs grown using Au catalyst particles by VLS mechanism.

XRD analysis has been carried out to characterize the silica NWs over larger area compared to the spatially confined TEM samples. The presence of Si nanocrystallites (as depicted in Fig. 4(b&c) of manuscript) is expected to result diffraction peaks for further validation. NWs growth over Si (100) samples with Au NPs in the range of 30-50 nm has been scanned over a wide 2θ range. As shown in above Fig. S4, the diffraction pattern is largely amorphous with diffraction peaks emerged from single crystalline Au catalyst particles from the tip of the NWs. Based on the TEM analysis, the observed Si crystallite are 6-10 times smaller than the catalyst particles. This results the observation of prominent diffraction from Au NPs compared to the embedded nano sized crystals.

S5: Growth rate estimate of silica NWs at 900ºC, Q_D-5*kT* and 10*kT:*

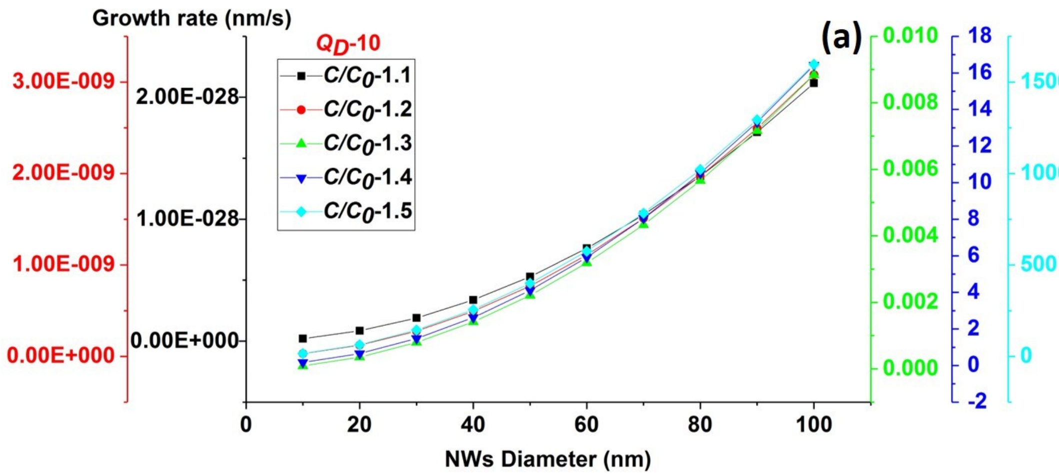

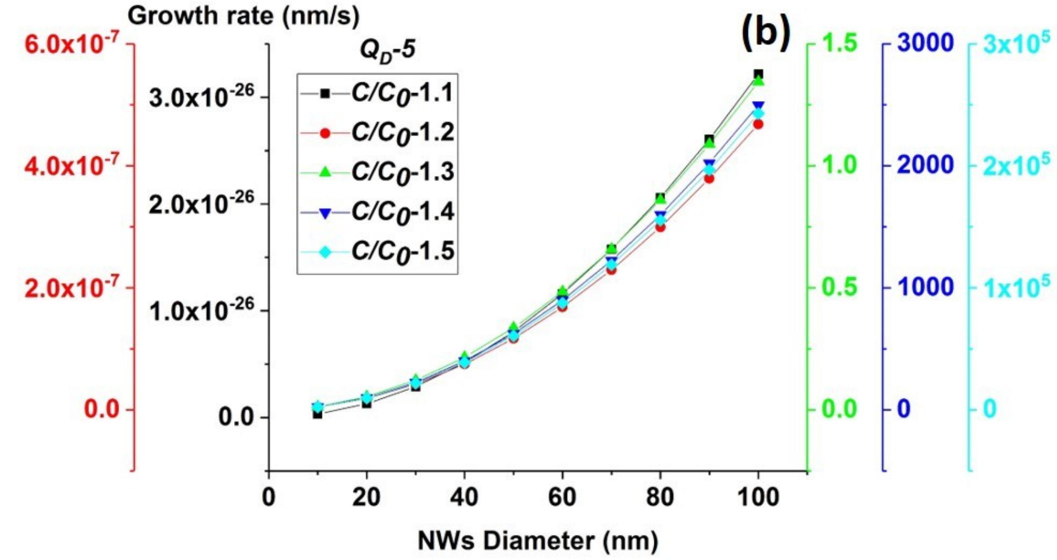

Fig.S5 (a). Crystallization rate of silicon at the interface at 900˚C with range of C/C^0 values and desolvation energy (Q_D) of 10*kT*. (a)2D plot showing that the crystallization rate spans several orders of magnitude for change of C/C^0 from 1.1-1.5.(b) Crystallization rate for Q_D-5*kT.*

S6: Growth rate estimate of silica NWs at 1000ºC, Q_D-10 *kT* :

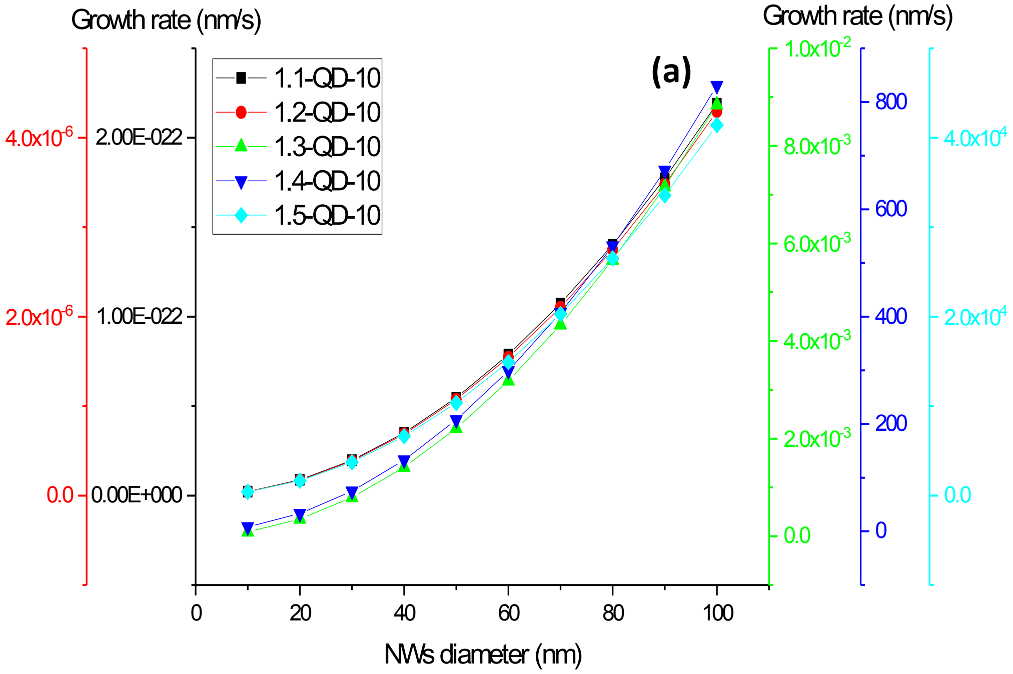
Growth rate (nm/s)
Growth rate (nm/s)
(a)
1.1-QD-10
1.2-QD-10
1.3-QD-10
1.4-QD-10
1.5-QD-10
4.0x10-6
2.0x10-6
0.0
2.00E-022
1.00E-022
0.00E+000
1.0x10-2
8.0x10-3
6.0x10-3
4.0x10-3
2.0x10-3
0.0
800
600
400
200
0
4.0x104
2.0x104
0.0
0
20
40
60
80
100
NWs diameter (nm)

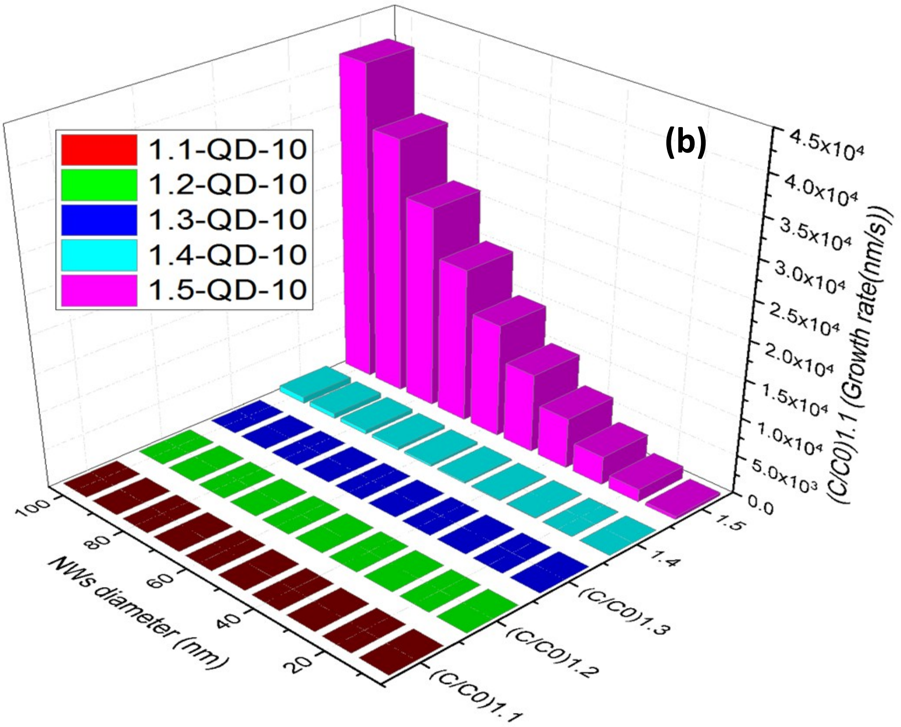
(b)
1.1-QD-10
1.2-QD-10
1.3-QD-10
1.4-QD-10
1.5-QD-10
4.5x104
4.0x104
3.5x104
3.0x104
2.5x104
2.0x104
1.5x104
1.0x104
5.0x103
0.0
(C/C0)1.1 (Growth rate(nm/s))
100
80
60
40
20
NWs diameter (nm)
(C/C0)1.1
(C/C0)1.2
(C/C0)1.3
1.4
1.5

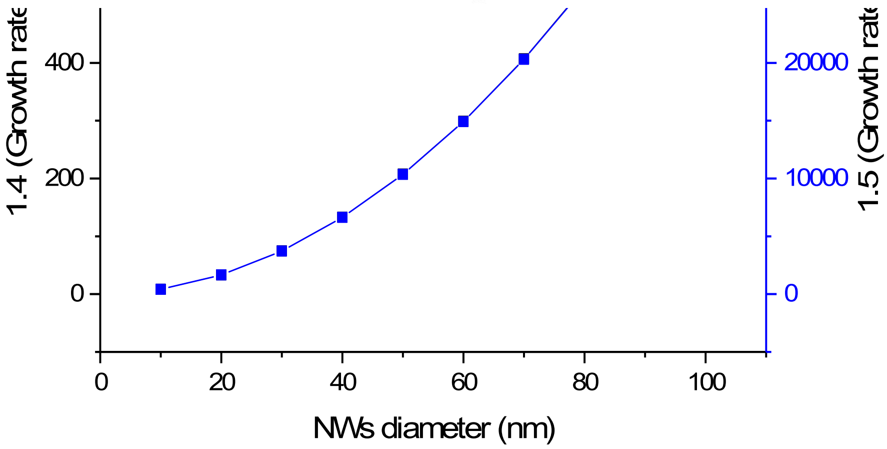
1.4 (Growth rate
1.5 (Growth rate
400
200
0
20000
10000
0
0
20
40
60
80
100
NWs diameter (nm)

Fig.S6 (a). Crystallization rate of silicon at the interface at 1000˚C with range of C/C^0 values and desolvation energy (Q_D) of 10*kT*. (a)2D plot showing that the crystallization rate spans several orders of magnitude for change of C/C^0 from 1.1-1.5.(b)3D plot of Si crystallization rates (c) Separated plots for 1.4 and 1.5 falls in the range of experimentally observed rates.

S7: Growth rate estimate of silica NWs at 1000ºC, Q_D-5kT:

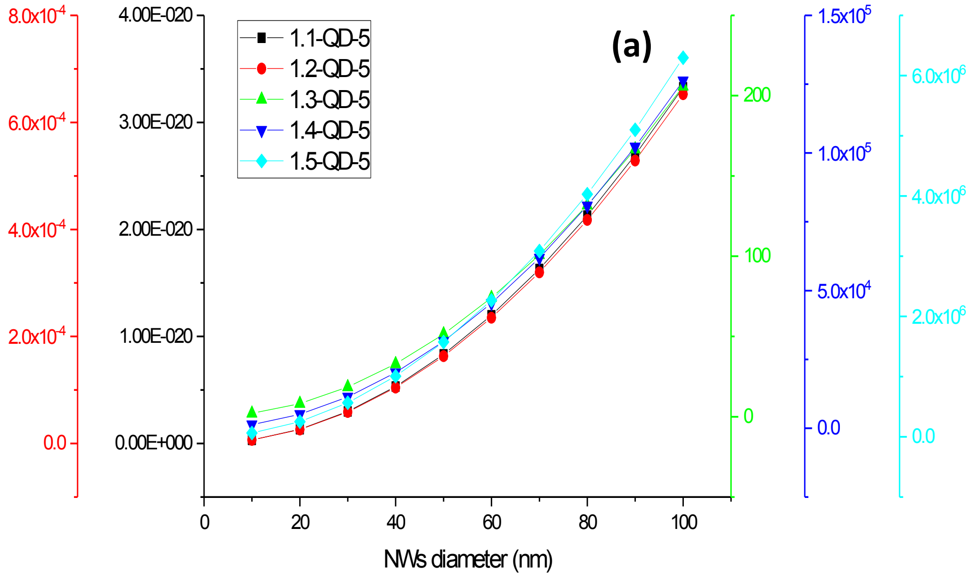

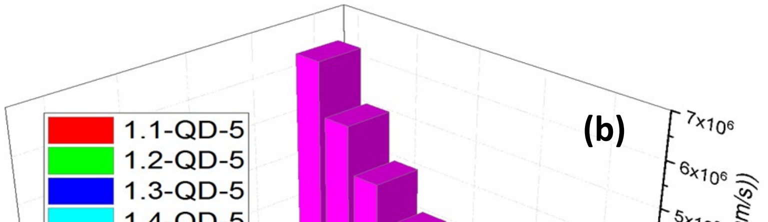

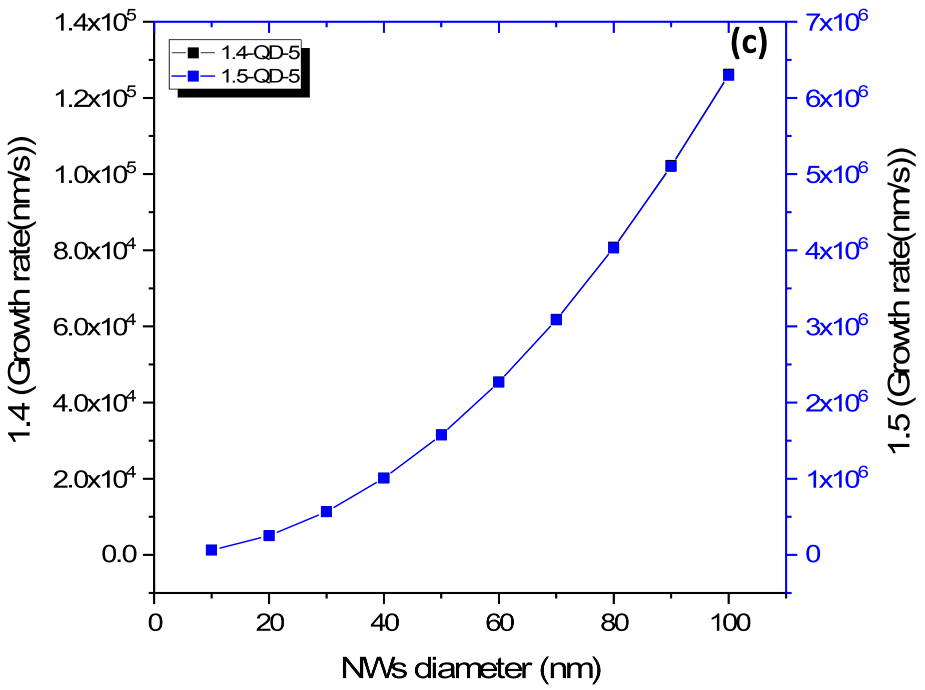

Fig.S7. Crystallization rate of silicon at the interface at 1000˚C with range of C/C^0 values and desolvation energy (Q_D) of 5kT. (a)2D plot showing that the crystallization rate for C/C^0 from 1.1-1.5.(b)3D plot of Si crystallization rates (c) Separated plots for (C/C^0) 1.4 and 1.5 falls in the range of experimentally observed rates.

S8:Experimental measurement of silica NWs growth rate:

The experimentally measured growth rate was found to closely match with the kinetic model estimation within the window of used physical chemistry parameters. This is illustrated below in Fig.S8 (a) and (b). Fig.S8a shows the measurement of NWs length with growth duration between 2-60 minutes. The major difficulty lies in the entanglement of the NWs which affects the accuracy of measurement. The two possible methods of measurements are, (1) Dispersion of NWs over a surface and carry out the microscopy length measurements. (2) Measurement using as grown substrate. Method (1) involves mechanical removal and solution dispersion of NWs which further affects the accuracy of measurement. Here we have used cross sectional SEM images (method 2) of Silica NWs (diameter between 20-75 nm) grown for various durations. The measurements have been carried out using ImageJ^{R} software tool. NWs growth process is generally classified by non-steady and steady state regions. A steady state region, at which the length of NWs are roughly proportional to the growth duration (Fig.S8a). The estimation of slope in steady state region (growth duration between 15-60 minutes) provides reliable growth rate of silica NWs. Multiple cross sectional SEM images have been used in these measurements. For example the statistical average length of 15 minutes grown NWs is 2780 nm with standard deviation of 705 nm. From these measurements , the estimated growth rate is 650 nm/s. Given the entanglement of the NWs, this could be the lower bound growth rate of silica NWs at 900˚C. Due to the aforementioned difficulties, there are no reports available with silica NWs growth rate measurement in the literature. The comparison of this measured growth rate with the kinetic model estimated values is presented in Fig.S8b. At temperature of 900˚C, the experimental measurement matched with the kinetic model parameters C/C_0-1.5 and Q_D -10. The measured growth rates are clearly demonstrated the validity of the kinetic parameters window chosen to estimate the growth rate of NWs. This will help to extend the applicability of the kinetic model towards other semiconducting oxide NWs.

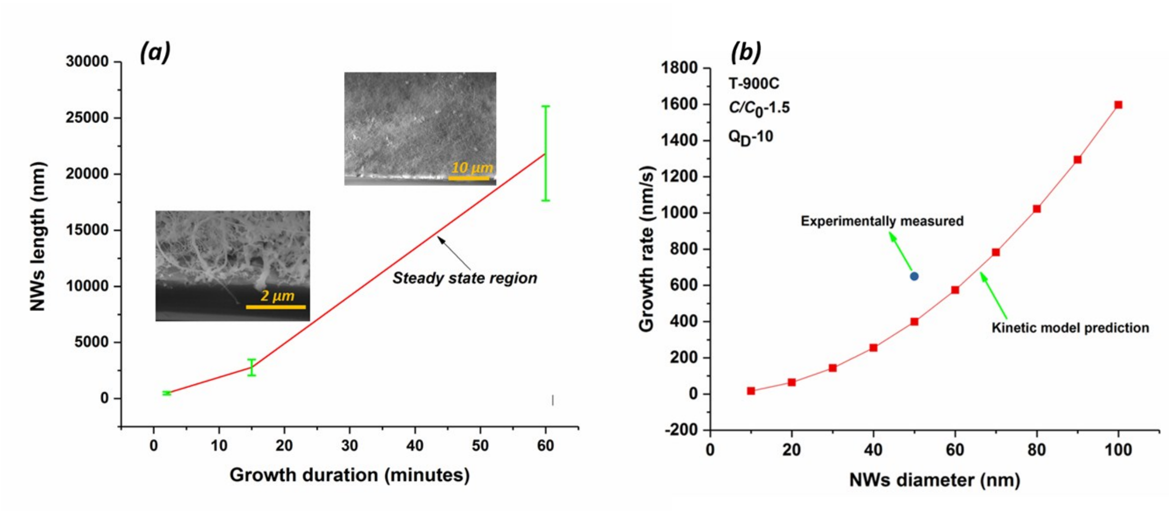

Fig.S8. Growth rate measurement and comparison with kinetic model estimated value. (a) Variation of NWs length with growth duration between 2-60 minutes. Inset shows the cross sectional SEM images used to measure the NWs length. (b)Comparison of experimentally measured growth rate versus kinetic model estimation.

S9:Statistical analysis of SiO_x and GeO_x NWs:

The statistical analysis of the silica NWs and GeOx NWs are presented below in Fig.S9. The measurement has been carried out using multiple SEM and bright field TEM images. ImageJ^R software tool was used to carry out these measurements. This study revealed two important aspects of the NWs growth by VLS mechanism. (1) The diameter of the NWs were in the size range of catalyst particle diameters. (2). The NWs were in uniform diameter at large length scale. As described in the experimental section, Au thin films of thickness in the range of 2-5 nm has been used to obtain NWs of diameter in the range of 10-100 nm in diameter. Typically, less than 3 nm films would results in sub-50 nm sized nanoparticles. However, other parameters such as temperature, annealing (or growth) duration, heating rate etc., has mild influence on the statistical distribution of the particles. The histograms in Fig.S9 shows that the NWs diameter distribution is falls well in the range of the kinetic study. These NWs has been used for the measurement of growth rate addressed in the previous section S8.

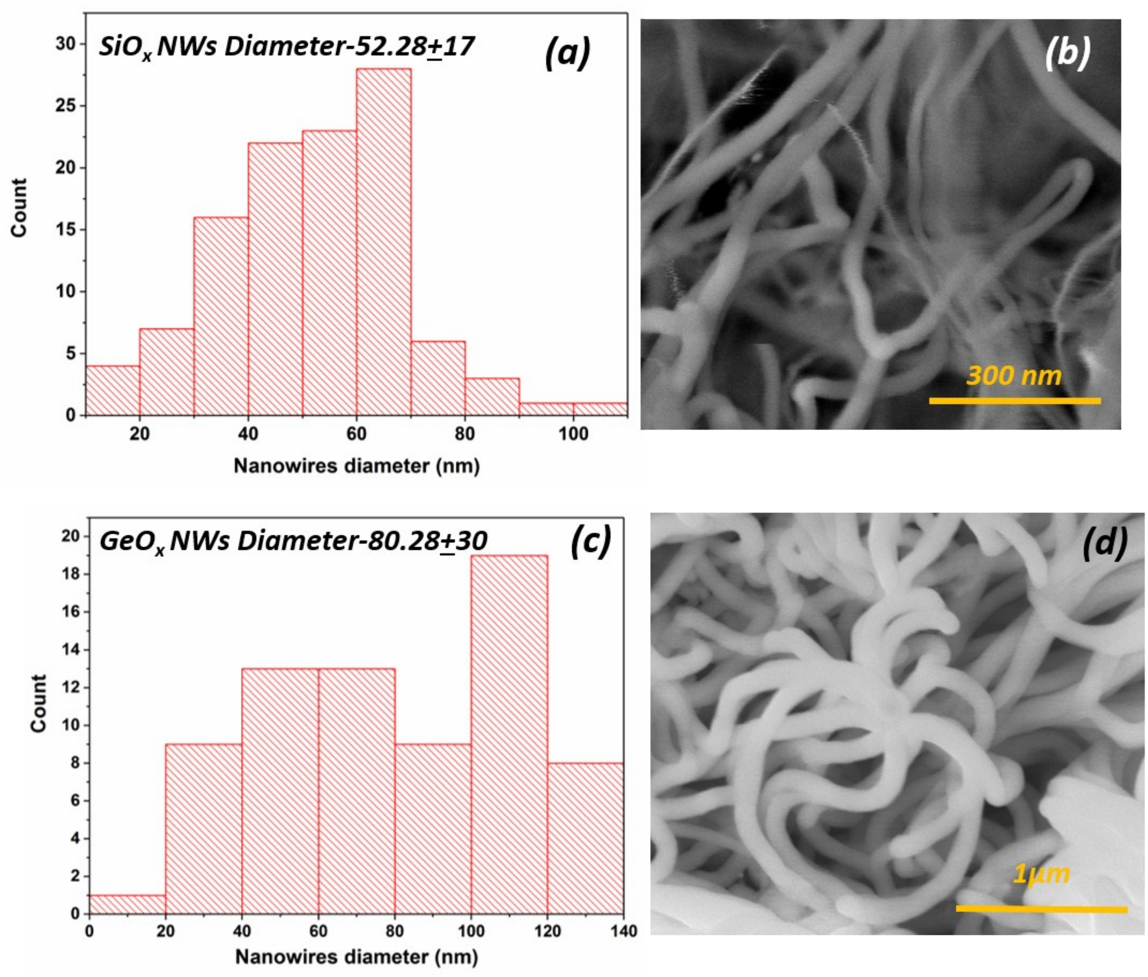

Fig.S9. The statistical distribution measurement of the SiO_x and GeO_x NWs in the ~ 100 nm diameter size range. (a&b). Silica NWs with a representative SEM image. (c&d). Measurement and analysis of GeO_x NWs.

S10:Oxidation rate of crystallized Si layers:

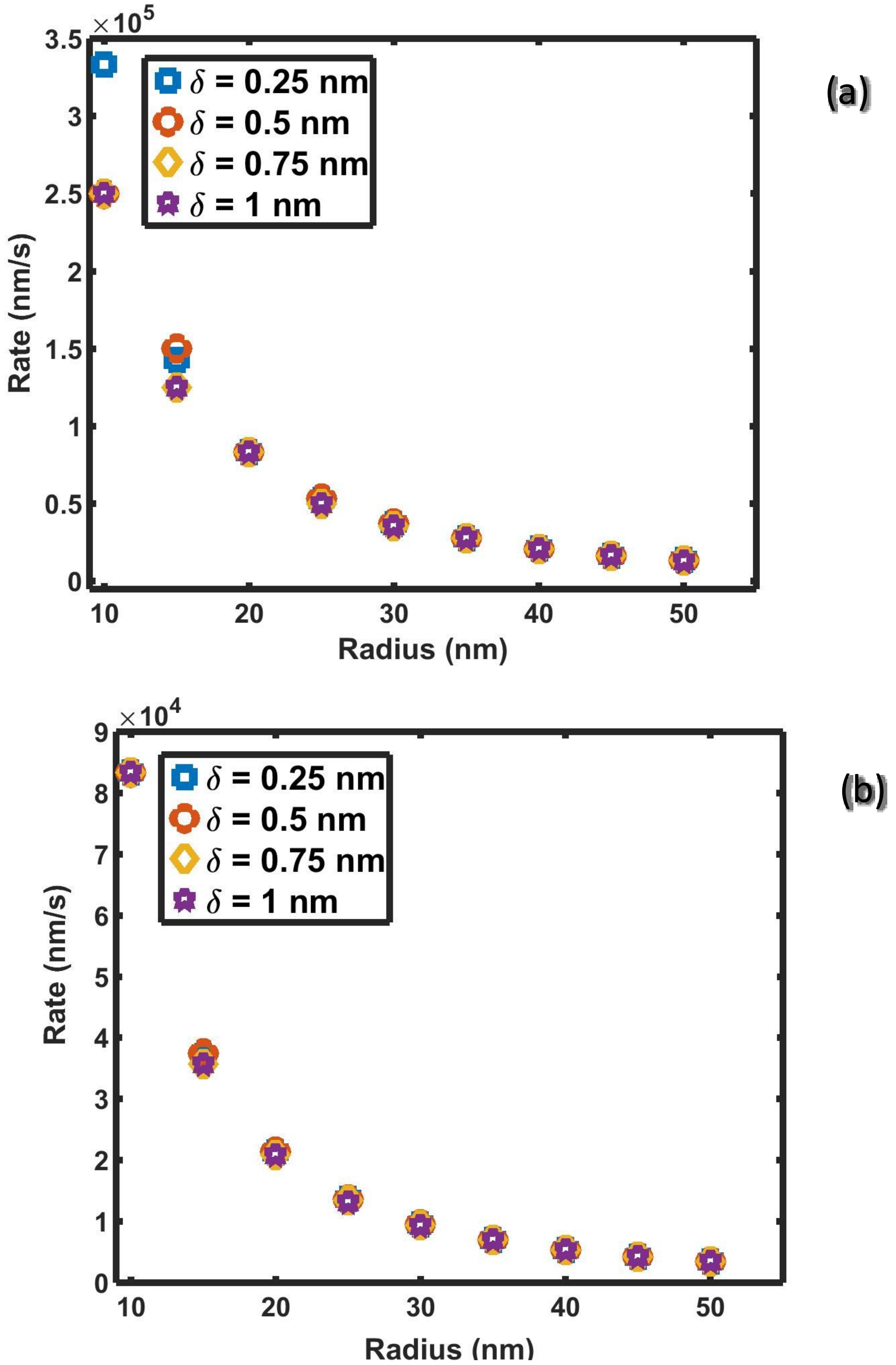

Fig.S10. Oxidation rate of crystallized Si layers for saturation fraction (a) 0.75 and (b) 0.99. The interface diffusion co-efficient 1.1×10^{-20} cm^2/s.
